# Adverse selection and consumer inertia: empirical evidence from the Dutch health insurance market

**DOI:** 10.1007/s10198-024-01725-8

**Published:** 2024-10-01

**Authors:** Ramsis R. Croes, Frederik T. Schut, Marco Varkevisser

**Affiliations:** 1 Dutch Healthcare Authority (NZa), Utrecht, The Netherlands; 2 Erasmus Centre for Health Economics Rotterdam (EsCHER), Erasmus University Rotterdam, Erasmus School of Health Policy and Management (ESHPM), Rotterdam, The Netherlands

**Keywords:** Health insurance, Adverse selection, Choice persistence, Consumer inertia, Deductible, G22, I13, I18

## Abstract

This paper examines to what extent consumer inertia can reduce adverse selection in health insurance markets. To this end, we investigate consumer choice of deductible in the Dutch health insurance market over the period 2013–2018, using panel data based on a large random sample (266 k) of all insured individuals in the Netherlands. The Dutch health insurance market offers a unique setting for studying adverse selection, because during annual open enrollment periods all adults are free to choose an extra deductible up to 500 euro per year. By focusing on deductible choices of those who do not switch health plans, we are able to examine the ‘pure’ adverse selection effect (i.e., not distorted by other health plan attributes). We estimate a dynamic logit model to examine individuals’ deductible choice. We find evidence of adverse selection, as people with higher previous health care cost are substantially less likely to take up or keep a 500-euro deductible. We also find that adverse selection is counteracted by a high level of consumer inertia, as the average partial effect on deductible choice of the previous selected deductible level is much larger than the average partial effect of a change in health care costs.

## Introduction

The presence of adverse selection is a well-known impediment to an efficient health insurance market [12]. Adverse selection occurs when enrollees choose health plans with more coverage because they have private information about being likely to incur high costs. Rothschild and Stiglitz [26] show that adverse selection can result in the underinsurance of low-risk enrollees or even in a market with no equilibrium. However, as shown by Handel [16] and Handel and Kolstad [17], adverse selection may in practice be counteracted by individuals’ suboptimal decision making. There is ample empirical evidence that optimal consumer choice in health insurance markets is hampered by “frictions” like inertia, search and switching cost, and a lack of knowledge (“health insurance literacy”); e.g., Samuelson and Zeckhauser [27], Abaluck and Gruber [1], Bhargava et al. [6], Handel [16], Handel and Kolstad [17], Handel et al. [19], Handel et al. [18], Heiss et al. [20], Ho et al. [21], and Marzilli Ericson [22].

Beforehand, it is not clear what is more important to market outcomes: adverse selection or frictions? The interaction between both potential market distortions is also unclear. As Pauly [24] noticed already four decades ago: “One of the things that theory does say here is that only a little bit of adverse selection may cause market equilibrium to unravel. But then only a little bit of consumer inertia is needed to reinstate it.”

The interaction of adverse selection and consumer inertia has recently been studied in the context of US markets for health insurance [16, 17, 19, 25]. Handel and Kolstad [17] measure inertia as the implied monetary costs of switching plans when a default option is present. They identify inertia by comparing health plan choice of the same consumers over time in both clearly active and clearly passive choice environments. In the context of an employment-based insurance setting of a large US firm, they show that both adverse selection and inertia are important. Furthermore, they show that reducing frictions is welfare decreasing (increasing) when the mean and variance of surplus from risk protection compared to its costs are relatively low (high).

In this paper, we aim to empirically determine to what extent adverse selection is mitigated by consumer inertia. In our setting—the Dutch market for mandatory basic health insurance—the choice environment is stable, since we focus on individuals staying with the same health plan during the study period. These individuals, however, can each year freely adapt their choice of deductible (i.e., coverage level) resulting in a lower or higher premium. Hence, no health plan attributes other than the deductible level and its corresponding premium difference play a role in the consumer choices examined here. Given that only monetary trade-offs are involved in this choice setting, we are able to identify inertia precisely as defined by Handel [16]: the implied monetary costs of choice persistence. Using detailed data for a large random sample (6 million) of the total Dutch adult insured population (approx. 14 million), we constructed a study sample consisting of about 1.8 million individuals who in the period 2013–2018 (i) did not switch health plans, (ii) each year had either a zero- or 500-euro deductible, and (iii) did not suffer from a severe mental illness. First, for these individuals we constructed 32 possible deductible choice paths to examine the relationship between health care costs and deductible choice. Second, we estimated a dynamic logit model using—for computational reasons—a smaller random subsample of people (about 266 k individuals). The model estimates reveal to what extent individuals’ previous and future health care costs impact their annual choice of deductible. Although we find clear evidence of adverse selection, we also find that this effect is strongly mitigated by the presence of substantial consumer inertia.

The remainder of this paper is organized as follows. In the next section, we briefly describe the context of the Dutch health insurance market in which people annually have free choice of deductible. Sect. "Methods and data" describes the methods, the empirical model and data. In Sect. "Results" the results are discussed. First the descriptive results showing the 32 possible deductible choice paths followed by the large study sample are analyzed. Then the estimation results for the empirical model for the subsample are presented. In Sect. "Conclusion and discussion" the main findings are summarized and briefly discussed.

## Context

In the Netherlands, universal mandatory health insurance is offered by competing private health insurers.^1^ During our study period (2013–2018) the number of basic health plans (or health insurance policies) offered by insurers varies between 55 and 67. All Dutch citizens are required to buy a basic health plan and health insurers are required to accept all individuals applying for enrollment. The basic benefit package is comprehensive and standardized by law. Hence, each basic health plan covers the same benefits. In addition, health plan premiums must be community-rated. That is, all people enrolling in the same health plan face the same premium (except that, during the study period, in case of a group contract insurers are allowed to offer a premium discount up to 10%).^2^ For all adult enrollees (18 years and older) there is a mandatory deductible. The level of this deductible is annually set by the government.^3^ On top of this mandatory deductible, adults can opt for an extra deductible in return for a premium discount. The voluntary deductible levels are restricted by the government to zero, 100, 200, 300, 400 or 500 euro per year. For each deductible level, health insurers are free to determine a community-rated premium discount. Expenses on maternity care, district nursing and family care (provided by GPs) are exempted from both the mandatory and voluntary deductible.

Each year, individuals can switch health plans during the six-weeks annual open enrollment season (mid-November until the end of December). Health plans differ from each other in terms of premium, service level, (preferred) provider network and premium discount for the various deductible levels. Enrollees can adjust the deductible level every year by notifying their health insurer during the open enrollment period. This typically requires only one phone call or ticking another box at the insurer’s website. Changing deductible levels does not require changing health plans. Hence, after having increased the deductible level people can easily lower it again during the next open enrollment season if they have acquired a chronic disease or otherwise expect higher medical costs in the year(s) to come.

## Methods and data

### Theoretical framework

In this paper, we aim to disentangle the impact of adverse selection and inertia on deductible choice by exploiting a unique feature of the Dutch health insurance market, i.e., individuals can annually change their deductible at virtually no switching cost without having to change health plans. Hence, for people sticking with their health plan but choosing a higher (lower) deductible only the level of financial protection decreases (increases) in return for a lower (higher) community-rated premium. Although we cannot observe individuals’ private information about their health risk, we do observe people’s health care expenses over the years. We therefore use people’s experience about their health expenses in the previous year as a proxy for the private information they possess about their health risk when having to choose a deductible in the current year. By focusing only on the deductible choices made by people who stick with their health plan, we rule out the possibility that observed deductibles choices are influenced by other plan characteristics. Hence, this approach allows us to isolate the potential adverse selection occurring in this market.

Assuming that people’s health care expenses in the previous year can serve as a proxy for private information, we measure adverse selection by the effect of people’s health care expenses in the previous year on their deductible choice in the current year (ceteris paribus other personal characteristics that may affect choice). In line with Handel and Kolstad [17], we define inertia by the implied monetary costs of switching plans when a default option is present. Atherly et al. [2] found that beneficiaries in Medicare Advantage in the US have strong preferences for remaining with the same insurer and plan over time, though rates of switching between plans within insurer were substantially higher than between insurers. However, the high switching costs generally found in the literature (for an overview, see [2]) appear to be due to that fact that in these (mainly US) settings switching health plans typically involves changes in many plan characteristics. In our study setting, however, the only plan characteristic that may change is the deductible level and the related premium discount offered by the insurer. Hence, we measure inertia by the effect of the deductible level chosen in the previous year on deductible choice in the current year, taking into account the premium discount offered by the health plan for taking-up a 500 euro deductible (ceteris paribus other personal characteristics that may affect choice).

### Empirical model

Using individual level data over the period 2013–2018, we estimate a dynamic logit model to examine how deductible choice in year *t* is affected by health care costs and deductible choice in year *t-1*. With this model, we explicitly consider two types of confounding that are relevant for our analysis. First, using individual fixed-effects we control for unobserved time-invariant individual heterogeneity (e.g., differences in risk preferences, education, social economic status, and health literacy). Second, for all individuals we use information about their drug consumption in the past to capture any chronic diseases and illnesses to control for the confounding effect of the presence a chronic disease on deductible choice. Although the possibility of any other time-variant personal characteristic potentially affecting deductible choice cannot be completely ruled out,^4^ we believe our approach is sufficient to control for the most relevant personal traits. Therefore, the effect of the lagged cost variable provides evidence for adverse selection, whereas the effect of the lagged deductible choice variable captures choice persistence and thus consumer inertia.

Our dynamic logit model specifies the probability ${\pi }_{i}$ that individual *i* (*i* = 1,..,*I*) chooses a 500 euro deductible in year *t* as:

$${\pi }_{it}=\frac{exp\left({\mu }_{it}\right)}{1+exp\left({\mu }_{it}\right)}$$

where ${\mu }_{i}$ denotes the set of relevant characteristics for individual *i*, which can be specified as follows:

$${{\mu }_{i,t}=c}_{i}+\beta {deductible}_{i,t-1}+ \sum_{k=1}^{2}{\rho }_{k}{cost}_{k,i,t-1} +\pi {chronic}_{i,t-1}+ \sum_{k=1}^{2}{\varphi }_{k}{cost}_{k,i,t} {chronic}_{i,t-1}+ \tau {discount}_{it }+ {\epsilon }_{it}$$

The main variables of interest in our model are *cost*_*t-1*_ and the state dependent parameter *deductible*_*t-1*_*.* We expect that having a 500-euro deductible in *t–1* has a positive impact on the probability of choosing a 500-euro deductible in *t*. If adverse selection is present, we expect to find a negative relationship between the uptake of a 500-euro deductible in *t* and health care cost in *t-1*.

For each individual, we categorized the health care cost in *t*, given by ${cost}_{i, t-1}$, in three different groups (k = 0, .., 2): [0, 385), [385,885) and [885, +) euro. These groups reflect the different deductible levels: (i) the first group having health care cost below the mandatory deductible (i.e., 385 euro per year); (ii) the second group having costs above the mandatory deductible but below the maximum total deductible (i.e. 385 + 500 = 885 euro), and (iii) the third group having costs above the maximum total deductible.^5^ In the model, the cost groups are included as dummy variables with the [0, 385] group as reference group. To check if our estimation results are sensitive to this categorization of costs, we also estimate a model with the continuous variable *log(cost* + *1)* in year *t-1* as predictor.

To control for the confounding of the presence of a chronic disease, we included a dummy variable equal to one if the enrollee had a chronic illness in year *t-1* and zero otherwise. In addition, to take into account the potential impact of differences in price, we include for each individual the community rated premium *discount* offered by his insurer for choosing a 500-euro deductible.

Ideally, we would like to estimate our dynamic logit model with individual time-invariant effects to control for unobserved time-invariant individual heterogeneity. However, we cannot estimate the model like the unconditional fixed effects maximum likelihood estimator with a dummy variable for each individual. Next to being computational costly, this will also be an inconsistent estimator due to the incidental parameters problem [28]. This refers to the following inference problem: as the number of individuals goes to infinity, the number of ${c}_{i}$’s (called incidental variables) also goes to infinity. As a result, the incidental variables will be inconsistently estimated, which contaminates the estimation of the other variables [23]. There are several model specific solutions (i.e. estimation methods) to the incidental parameters problem, but not a unified one [8].

Note that in linear models, we could easily estimate a panel model with individual fixed effects by means of first differencing or using a within transformation (where the ${c}_{i}$’s are eliminated). However, since the binary outcome panel model is nonlinear, estimating a linear probability model may result in predicted probabilities outside the [0, 1] interval. Furthermore, estimating a fixed effects model in our case is biased due to the incidental parameters problem mentioned above. Therefore, we prefer the non-linear approach outlined above.

There are several bias corrections available to reduce the bias due to the incidental parameters and methods for reducing the computational costs (e.g., [14, 15]). However, bias correction methods could be computational costly. For our estimation, we therefore used a recently developed estimation technique: the pseudo conditional maximum likelihood (PCLM) estimator developed by Bartolucci and Nigro [4]. The PCLM estimator approximates the dynamic logit model through a quadratic exponential model. It follows a similar approximation approach as Bartolucci and Nigro [3]. Using this estimator, the incidental parameters problem is solved by conditioning on sufficient statistics for the individual intercepts, which are based on the sums of the response variable on the individual level.

Bartolucci and Nigro [4] performed simulations to determine the finite sample properties of their pseudo estimator. Their simulations showed that the estimator has a has a very low bias for the covariates and the state dependence, indicating that it performs well. Furthermore, they showed that their estimator, compared to alternative estimators, usually has a smaller bias and a greater efficiency.

### Data

We use individual level panel data, obtained from the Dutch Healthcare Authority (NZa), covering the entire Dutch adult population (approx. 14 million people) between 2013 and 2018. The dataset includes information on (i) each person’s health care expenses, including out-of-pocket costs, for benefits covered by mandatory health insurance, and (ii) each person’s choice of health plan and deductible level. In addition, we also obtained data from the NZa about the community-rated premium discount offered by each health plan in return for a higher deductible. Throughout the study period, the average premium discount for a 500-euro deductible was 232 euro (min. 150 euro, max. 324 euro).

As a start, we took a random sample of 6 million individuals that were in the dataset for the year 2018. Next, we excluded individuals that were not in the dataset the whole study period (2013–2018) and those younger than 18 years in any of these years, since these individuals did not face any deductible. We also excluded a small number of people with more than one health plan per year (e.g., because they enrolled in another group contract after changing jobs), and people with incomplete information on health expenses. Both groups comprise less than 1% of the total population. The remaining final study sample includes about 3.3 million individuals with data for each of the six years between 2013 and 2018.

For this balanced panel, Table 1 shows the distribution of individuals over the various deductible levels. The proportion of enrollees that opted for a voluntary deductible other than zero varied between 9 and 12%. In any year, the most frequently chosen non-zero deductible was 500 euro.

**Table 1:** Distribution of enrollees over the voluntary deductible levels

	2013%	2014%	2015%	2016%	2017%	2018%
0 euro	91	90	88	89	89	90
100 euro	1	1	1	1	1	0
200 euro	1	1	1	1	1	1
300 euro	1	1	1	1	1	1
400 euro	0	0	0	0	0	0
500 euro	5	7	8	8	8	8

In our dataset, 38% of all enrollees who changed their deductible, also switched health plans. The decision to switch health plans depends on multiple factors [7]. Since these factors may be correlated with the choice of deductible, we restrict our analysis to those enrollees that did *not* switch health plans during the study period (but who might have changed their choice of deductible). For this subsample, it is most likely that choices for another deductible level are driven by past or anticipated health care expenses. Furthermore, to keep the analysis concise we also excluded the small minority of people (2–4%) who chose intermediary deductible levels (100–400 euro). Lastly, we also excluded individuals with severe mental illness since they are unlikely to make deliberate choices concerning their health insurance.^6^

The final sample consists of about 1.8 million individuals who in the period 2013–2018 (i) did not switch health plans, (ii) each year had either a zero or a 500-euro deductible, and (iii) did not suffer from a severe mental illness. Compared to the overall Dutch population, the enrollees included in our selection are somewhat older, more likely to have a chronic disease, less likely to have a 500-euro voluntary deductible and have higher health care costs. As explained in Sect. "Theoretical framework", we do not claim that our sample is representative for the overall population because using this selection of enrollees allows us to isolate the potential adverse selection occurring in the Dutch health insurance market.

## Results

### Descriptive results

In our regression, we use people’s health care expenses in previous years for explaining their deductible choice in year *t.* Therefore, we lose one year of deductible choices (2013) in our analyses. For this reason, we limit the descriptive analyses to the years 2014–2018. Given this 5-year study period and the two deductible choice options considered (0 or 500 euro), 32 possible deductible choice paths can be distinguished (see Appendix I). These paths will be discussed in more detail below.

**Fig. 1:**
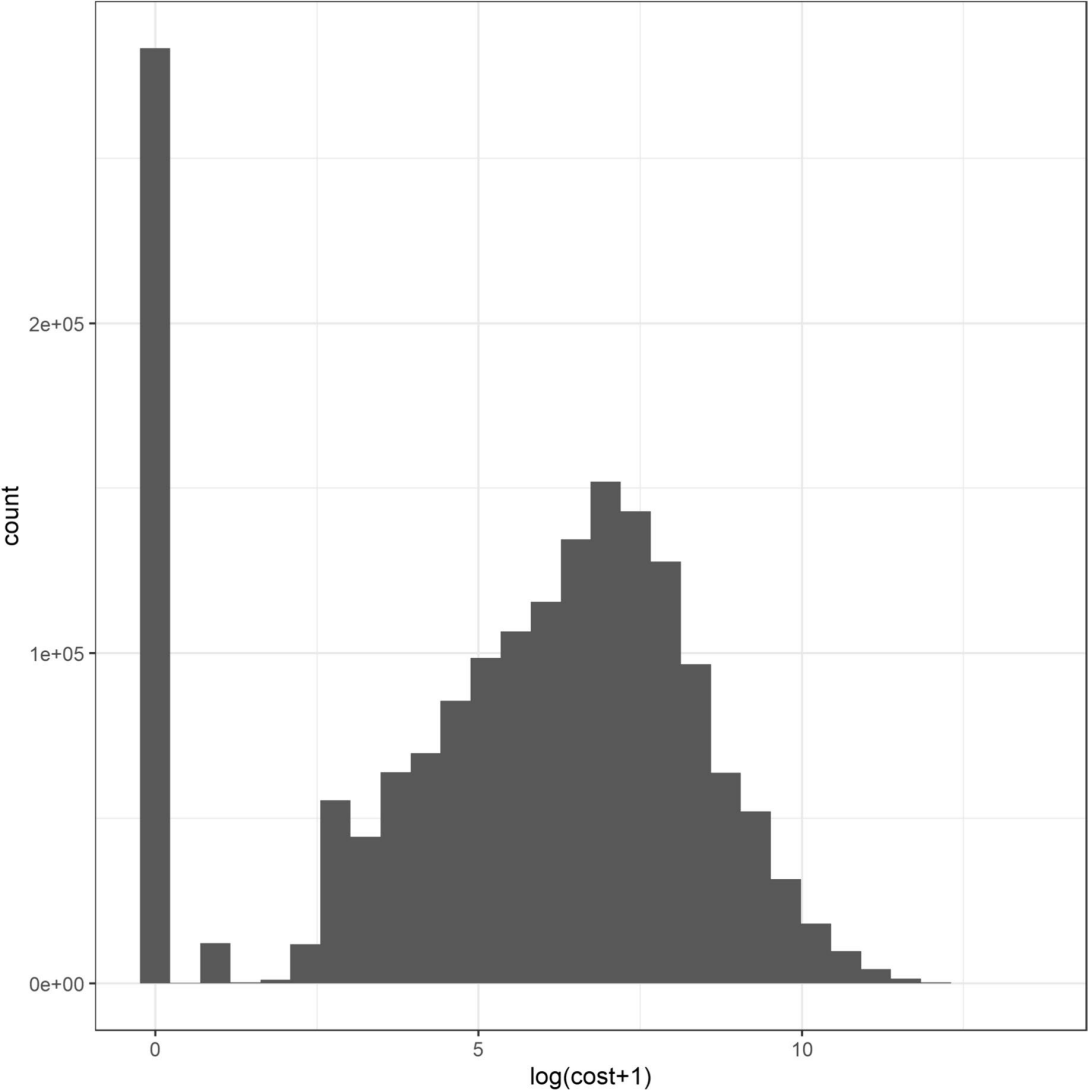
Distribution of *log(cost* + *1)* transformed individual health care expenses

When calculating people’s health care costs, we excluded the costs of GP-care, district nursing and maternity care because these are exempted from the deductible. Since the distribution of individual health care costs is highly skewed, we transformed the cost data by taking the natural logarithm of one plus the costs, i.e., *log*(*cost* + *1*). Figure 1 shows that the resulting log transformed distribution has the familiar bimodal shape, with local maxima at 0–0.5 (≈ 0 euro) and 6.5–7 (≈ 900 euro).

Table 2 displays the proportion of people with either a zero- or 500-euro deductible in 2018 for 6 different cost categories. As expected, the share of people opting for the highest deductible in 2018 is negatively related to their health care expenses in the current year. In the lowest cost category 12.7% of the individuals opted for a 500-euro deductible, whereas this deductible was chosen by only 0.8% of the individuals in the highest cost category. However, even among those in the lowest cost category more than 87% of the individuals preferred not to opt for a voluntary deductible.

**Table 2:** People with a zero- or 500-euro deductible in 2018 per cost category

*log*(*cost* + *1*)	Actual costs ranges	People with deductible 0 euro	People with deductible 500 euro	People (%) with deductible 500 euro %
[0,2]	[€0,€6]	259,047	37,673	12.7
(2,4]	(€6,€54]	165,525	18,153	9.9
(4,6]	(€54,€402]	374,538	23,923	6.0
(6,8]	(€402,€2979]	582,151	13,753	2.3
(8,10]	(€2979,€22025]	274,449	2,712	1.0
(10,15]	(€22,025,€3269016]	33,679	256	0.8

The high percentage of people with low costs choosing a zero deductible indicates that many people may not make a (financially) optimal deductible choice. Indeed, as showed by Van Winssen et al. [32] an uptake of a 500 euro deductible would have been retrospectively financially profitable for 48% of the Dutch insured population in 2014, whereas only about 11% actually did so.^7^ In another study, using data for the years 2006–2013, Douven et al. [11] found that almost half of the people without a 500 euro deductible would have financially benefited if they had opted for this. They also found that for 80–90% of the people with a 500-euro deductible this was ex-post profitable. Similar results are presented by Handel et al. [18]. They found that in 2015 about 52% of all Dutch consumers would have been better off with a 500-euro voluntary deductible, while this was taken by less than 7%.^8^ Of course, an *ex-post* non-profitable deductible choice does not necessarily mean this choice is also non-profitable *ex-ante*, because the ex-ante profitability depends on the distribution of risk as well as people’s risk preferences.^9^

For each of the 32 deductible choice paths (see Appendix I), we examined the relation between healthcare costs and deductible choice more closely. More specifically, for each choice path we calculated the aggregate annual median healthcare costs. In Appendix I (Table 5), the resulting median cost patterns are presented.

As Table 5 shows, the vast majority (93%) of all enrollees included in the dataset sticks with the default option of a zero deductible during the entire period (path 1). The second largest subgroup (almost 4%) consists of enrollees who stick with a once chosen 500-euro deductible (path 32). The remaining 3% changed their deductible at least once during the study period.

As expected, enrollees sticking with a 500-euro deductible throughout the entire study consistently have the lowest annual median costs, whereas enrollees sticking with a zero deductible consistently have (one of) the highest median costs. These median cost patterns are consistent with the presence of adverse selection: high (low) risks seem to sort themselves into low (high) deductible plans.

Of particular interest are the choice paths of enrollees who experience a substantial increase in health care expenses over the years. For many choice paths in which enrollees experience a strong increase in costs—e.g., paths 29 and 31—we observe that the cost jump is followed by a change in deductible from 500 to zero in the next year. This is consistent with the presence of an adverse selection effect. Enrollees following choice path 22, 24, 28, and 30 substituted a zero for a 500-euro deductible in the only year(s) they had higher health care cost and immediately changed back to a zero deductible in the next year if their health care costs had returned to a low level. This is consistent with both adverse selection (choosing a zero deductible in anticipation of health care expenses) and moral hazard (a zero deductible resulting in higher health care expenses). Choice paths 2, 4, and 8 show that enrollees with a zero deductible who are experiencing stable low health care costs over the entire period eventually opted for a 500-euro deductible. This demonstrates that adverse selection does not necessarily take place immediately because over time consumers may learn more about their health risk, and the corresponding health care expenses, as well as about the available deductible choice options.

In sum, all the possible choice paths seem to have cost patterns that are, at least to some extent, consistent with the presence of adverse selection. The patterns also suggest that (i) adverse selection may take time to arise, and (ii) some healthy people are able to effectively anticipate an increase in next year’s health care costs.

### Estimation results

As explained above, we used the PCLM estimator [4] for estimating our dynamic logit model. For computational reasons, we took a random sample of 500,000 individuals from our study sample. Subsequently, we selected individuals who are (i) 18 years and older, (ii) did not switch health plan and (iii) did not have a chronical mental illness.^10^ This selection reduced our final sample to 265,629 individuals. For these individuals three specifications of our model were estimated. In the first and second specification costs are included as dummy variables based on the maximum (mandatory and voluntary) deductible people have to pay out-of-pocket, i.e., people with healthcare cost in one of the [0, 385), [385, 885) and [885, +) euro groups. Additionally, in the second specification, we also included interactions between these cost groups and the lagged dummy variable for being chronically ill (${chronic}_{i,t-1}$). As a sensitivity check, in the third specification health care costs are included as a continuous variable *log(cost* + *1)*. Table 3 gives the estimated coefficients for each of these three model specifications.

**Table 3:** Estimation results dynamic logit model

	*Dependent variable:* *500-euro deductible (1* = *yes/0* = *no)*		
	(1)	(2)	(3)
*Deductible*_*t-1*_	4.279** (0.091)	4.290** (0.091)	4.286** (0.091)
${cost}_{i, t-1}$[385,885)	− 0.406** (0.063)	− 0.426** (0.067)	
${cost}_{i,t-1}$[885, +)	− 1.266** (0.060)	− 1.139** (0.063)	
${\text{log}(cost}_{i, t-1}+1)$			− 0.152** (0.008)
${chronic}_{i,t-1}$		− 0.315 (0.184)	
${{cost}_{i, t-1}[\text{385,885})chronic}_{i,t-1}$		0.021 (0.213)	
$${{cost}_{i,t-1}[885,+)chronic}_{i,t-1}$$		− 0.844** (0.212)	
${discount}_{it}$	0.300** (0.055)	0.293** (0.055)	0.319** (0.055)

Note: ** = *p* < 0.01, * = *p* < 0.05 and standard error in parentheses

As expected, the coefficients for the lagged health care costs—(${cost}_{k,i,t-1}$) in specifications 1 and 2 or $\mathit{log}\left({cost}_{i,t-1}+1\right)$ in specification 3—are negative. Hence, higher costs in year *t-1* lower the probability of having a 500-euro deductible in year *t*, all else equal. The coefficient for having a 500-euro deductible in year *t-1* (${deductible}_{i,t-1}$) is positive. This implies that, as expected, already having a deductible of 500 euro increases the probability to choose it again, all else equal. It also implies that having no voluntary deductible in year *t-1* lowers the probability of choosing a deductible of 500 euro instead next year.

The premium discount (${discount}_{it}$) also has a positive coefficient, which implies that, as expected, people are more likely to choose a 500-euro deductible if they get a higher premium discount from the insurer. In model 2 we explicitly controlled for each individual’s health status by including the dummy variable capturing chronic diseases (${chronic}_{i,t-1}$) as well as its interactions with the cost categories (${cost}_{k,i,t} {chronic}_{i,t-1}$). Also conform to expectations, we find that people with a chronic disease are less likely to choose a 500-euro deductible. The coefficients for our main variables of interest (*cost*_*t-1*_ and *deductible*_*t-1*_) do not change and thus seem to be robust.

To obtain a better indication of the effects of the explanatory variables included in our model, we use the Average Partial Effect (APE). This measures the change in the expected outcome (i.e. the probability of choosing a 500-euro deductible) due to a small change in a covariate. However, if the number of periods is fixed, the APE of some covariate is generally biased. This is due to the incidental parameters problem since the estimation of the individual effects is biased, which also has an effect on the slope parameters [5].

As a solution, Bartolucci and Pigini [5] proposed an APE estimator that still has asymptotic bias but performs well in finite samples, even when *I* (number of individuals) is much larger than *T* (number of periods). Moreover, the bias corrected estimate of the unobserved heterogeneity entails a substantial improvement over the standard ML estimate with short *T*. Let vector ${{\varvec{w}}}_{itk}$ collect all model variables. Following Bartolucci and Pigini [5], the partial effect of covariate *k* for individual *i* at period *t* is defined as

$${v}_{itk}\left({c}_{i},{\varvec{\theta}}, {{\varvec{w}}}_{it}\right)=\left\{\begin{array}{l}p\left({\pi }_{it}= 1|{c}_{i}, {{\varvec{w}}}_{it}\right)\left[1 - p\left({\pi }_{it} = 1|{c}_{i}, {{\varvec{w}}}_{it}\right)\right]{\delta }_{k,}\\ with \,{w}_{itk} \,continuous\\ p\left({\pi }_{it} = 1|{c}_{i}, {{\varvec{w}}}_{it,-k,}{w}_{itk}=1\right)-\left({\pi }_{it} = 1|{c}_{i}, {{\varvec{w}}}_{it,-k,}{w}_{itk}=0\right),\\ with\, {w}_{itk} \,discrete\end{array}\right.$$

where ${{\varvec{w}}}_{it,-k}$ denotes the vector with ${{\varvec{w}}}_{it}$ excluding ${{\varvec{w}}}_{itk}$, and vector ${\varvec{\theta}}$ collects all model coeficients.

The APE of covariate *k* can be estimated by

$${\widetilde{v}}_{k}=\frac{1}{nT}\sum_{i=1}^{I}\sum_{t=1}^{T}{v}_{itk}\left({\widetilde{c}}_{i}\left(\widetilde{{\varvec{\theta}}}\right),\widetilde{{\varvec{\theta}}},{{\varvec{w}}}_{it}\right),$$

where $\widetilde{{\varvec{\theta}}}$ are the above estimated coeficients and Bartolucci and Pigini [5] uses the modified score function by Firth [13] to estimate ${\widetilde{c}}_{i}\left(\widetilde{{\varvec{\theta}}}\right)$. See Bartolucci and Pigini [5] for the calculation of the standard errors for ${\widetilde{v}}_{k}$ 11.

Table 4 presents, for each model specification, the estimated APEs including the standard errors. From these effects it can be concluded that having chosen a 500-euro deductible in year *t-1* increases the probability to choose this deductible in year *t* with approximately 70 percentage points in all three model specifications. This indicates the presence of a large choice persistence effect.

**Table 4:** Average Partial Effects (APEs)

	*Dependent variable:* *500-euro deductible (1* = *yes/0* = *no)*		
	(1)	(2)	(3)
*deductible*_*t-1*_	0.707** (0.006)	0.708** (0.013)	0.702** (0.006)
${cost}_{i,t-1}$[385,885)	− 0.051** (0.008)	− 0.053** (0.008)	
${cost}_{i,t-1}$[885, +)	− 0.157** (0.007)	− 0.142** (0.008)	
${\text{log}(cost}_{i,t-1}+1)$			− 0.019** (0.001)
${chronic}_{i,t-1}$		-0.040 (0.023)	
${{cost}_{i,t-1}[\text{385,885})chronic}_{i,t-1}$		0.003 (0.027)	
$${{cost}_{i,t-1}[885,+)chronic}_{i,t-1}$$		− 0.105** (0.026)	
${discount}_{it}$	0.038** (0.007)	0.037** (0.007)	0.040** (0.007)

Note: ** = *p* < 0.01, * = *p* < 0.05 and standard error in parentheses

In model specification 1 and 2, people in *cost_cat[385,885)* in year *t-1* have in year *t* a 5.1 and 5.3 percentage points lower probability to choose a 500 deductible when compared to people in the reference cost group *cost_cat[0,385)*. For people in *cost_cat[885,* +*)* this decrease in probability is 15.7 and 14.2 percentage points respectively.

In model specification 3, an increase of 10% in *log(cost* + *1)* in year *t-1* reduces the probability of of choosing a 500-euro deductible in year *t* with 1.9 percentage points. As an illustration, a 10% increase in log health care costs for example reflects an increase from exp(6) = 403 euro to exp(6.6) = 735 euro.

Looking at the interactions in model specification 2, only the interaction between having expenses in *cost_cat[885,* +*)* in *t-1* seems relevant. Having expenses in the highest cost category and having a chronic illness in *t-1* gives in *t* an additional reduction in the probability of choosing a 500-euro deductible of 11 percentage points on top of the 14 percentage points decrease when having high cost without having a chronic illness in *t-1*. This suggests that people are much more likely to opt for a lower deductible when high costs are associated with a chronic illness than when high costs are caused by non-chronic diseases.

Overall, from our estimation results we conclude that in all model specifications our estimated APE indicates large choice persistence. However, we also find evidence of substantial adverse selection since significant number of people incurring high healthcare expenses and/or a chronical condition in year *t-1* are likely to reduce their 500-euro deductible to zero in year *t*.

## Conclusion and discussion

We used the unique context of the Dutch health insurance market, where people can annually choose for an extra voluntary deductible varying from 0 to 500 euro without having to change health plans, for quantifying the opposing effects of consumer inertia and adverse selection. Using data for a large random sample (6 million) of the total Dutch adult insured population (approx. 14 million), we constructed a study sample consisting of about 1.8 million individuals who in the period 2013–2018 did not switch health plans and each year had a zero- or 500-euro deductible.

For these people, we first examined the 32 possible deductible choice paths. This reveals that all choice paths have cost patterns that are consistent with the presence of adverse selection. The patterns also suggest that adverse selection may take time to arise since it can take several years before people with low health care cost substitute a 500-euro for a zero deductible. In addition, the choice paths show that on average healthy people can anticipate effectively next year’s health care costs.

Next, using a smaller random subsample of 266 k individuals, we estimated a dynamic logit model for examining to what extent the individual choice of deductible in year *t* can be explained by their deductible choice in year *t-1* as well as health care costs in year *t-1*.We find clear evidence of adverse selection, as people with higher previous health care costs are substantially less likely to take up or keep a 500 euro deductible. However, we also find clear evidence of high consumer inertia as the average partial effect of already having a deductible in year *t-1* is much larger than the average partial effect of changes in health care costs. The substantial degree of choice persistence is remarkable, given the very low transaction costs for enrollees involved in adjusting their deductible level and the implied monetary costs of choice persistence. The missed premium discount equals approximately 200 euros per person per year.

As shown by Handel et al. (2015) a certain degree of consumer inertia may be welfare increasing because it may effectively counteract adverse selection. In the Netherlands, this potential welfare improving effect of consumer inertia may be small, because adverse selection is already quite effectively mitigated by a sophisticated system of risk equalization [30]. Nevertheless, even after sophisticated risk equalization those opting for the highest deductible level still appear to be quite profitable to insurers at the prevailing premium discount levels [9]. Future research may reveal the extent to which improving consumer choice of deductibles in the presence of sophisticated risk equalization will indeed lead to welfare gains.

## Appendix I

Deductible choice paths

See Table

**Table 5:** Choice paths and annual median costs

	Voluntary deductible level (€)			Annual median costs (€)					
	2014_2015_2016_2017_2018	N	%	2013	2014	2015	2016	2017	2018
1	000_000_000_000_000	1,663,338	93.14	377	400	358	411	421	488
2	000_000_000_000_500	5262	0.29	69	63	49	44	36	38
3	000_000_000_500_000	865	0.05	142	126	111	78	263	434
4	000_000_000_500_500	5585	0.31	57	54	42	35	32	40
5	000_000_500_000_000	748	0.04	187	166	106	468	485	357
6	000_000_500_000_500	93	0.01	94	91	78	77	233	69
7	000_000_500_500_000	637	0.04	137	117	74	87	334	394
8	000_000_500_500_500	4831	0.27	55	49	35	30	31	39
9	000_500_000_000_000	1384	0.08	158	155	350	512	352	344
10	000_500_000_000_500	106	0.01	114	160	110	452	119	58
11	000_500_000_500_000	44	0.00	166	147	106	537	167	782
12	000_500_000_500_500	155	0.01	120	78	81	280	45	64
13	000_500_500_000_000	1072	0.06	130	115	99	404	508	301
14	000_500_500_000_500	183	0.01	114	84	53	131	440	77
15	000_500_500_500_000	961	0.05	114	87	57	92	363	511
16	000_500_500_500_500	8761	0.49	45	40	24	29	29	36
17	500_000_000_000_000	5609	0.31	125	478	451	338	299	313
18	500_000_000_000_500	184	0.01	78	255	464	131	87	68
19	500_000_000_500_000	80	0.00	106	305	430	197	544	724
20	500_000_000_500_500	301	0.02	84	153	487	130	38	81
21	500_000_500_000_000	104	0.01	120	313	562	550	888	471
22	500_000_500_000_500	30	0.00	76	44	656	99	438	48
23	500_000_500_500_000	100	0.01	72	145	374	59	450	778
24	500_000_500_500_500	623	0.03	46	92	411	39	48	56
25	500_500_000_000_000	4977	0.28	72	99	389	479	274	268
26	500_500_000_000_500	296	0.02	33	64	259	508	143	80
27	500_500_000_500_000	150	0.01	70	73	125	393	207	594
28	500_500_000_500_500	759	0.04	39	45	108	513	37	54
29	500_500_500_000_000	4508	0.25	59	66	85	539	489	302
30	500_500_500_000_500	778	0.04	50	45	48	149	552	74
31	500_500_500_500_000	4812	0.27	45	44	44	63	375	521
32	500_500_500_500_500	68,523	3.84	26	26	16	16	17	23
		1,785,859	100						

^5^

## Footnotes

1 For a more detailed description of the Dutch health insurance market and the system of regulated (or managed) competition, see Van de Ven and Schut [29], and Douven et al. [10].

2 For each enrollee insurers receive a risk-adjusted premium subsidy from a risk-equalization fund that is filled with income-related contributions. This risk-adjusted premium subsidy is equal to the enrollee’s predicted costs minus a fixed amount that is annually determined by the government (about 1,000 euro per year). Hence, to break even health insurers must at least charge a community-rated premium equal to this fixed amount [31].

3 During our study period the mandatory deductible was gradually raised from 350 euro in 2013 to 360 euro in 2014, 375 euro in 2015, and 385 euro from 2016 onwards.

4 Income may be a time-variant personal characteristic affecting deductible choice. Unfortunately, reliable data on (individual and household) income is lacking in the data. Given the relative short time frame of our study, however, differences in income across individuals are likely to be quite stable for most individuals and therefore largely captured by the fixed-effects.

5 We took the 2016 mandatory deductible (385 euro) as uniform cutoff point for all years. Although the mandatory deductible in 2013, 2014 and 2015 was slightly lower (350–375 euro) this difference was very small.

6 Specifically, we excluded individuals that were prescribed at least a certain amount of medication for bipolar or psychotic disorders, which are commonly defined as severe mental disorders. These individuals were identified by their classification in the relevant Pharmaceutical Cost Groups (PCGs), which are used as risk adjusters in the Dutch system of risk equalization.

7 Handel et al. [19] find similar differences in a US employment-based insurance context. Based on ex post spending, 60% of employees would have been better off financially in a high deductible health plan though only 15% of employees actually chose such a plan.

8 Based on their study, only 54% of the Dutch population chose the cost-minimizing deductible leaving on average 66 euro per individual on the table.

9 Note that the rationale of insurance is that ex-post the ‘lucky’ majority of the insured make a personal loss because their premiums are used to pay the claims of the ‘unlucky’ minority.

10 These ilnesses are psychosis, chronic mood disorder, substance use disorder, bipolar disorder, and attention deficit hyperactivity disorder.

11 Bartolucci and Pigini graciously provided the authors the R-code that calculates the APEs.
